# COVID-19 Lessons and Post-pandemic Recovery: A Case of Latvia

**DOI:** 10.3389/fpubh.2022.866639

**Published:** 2022-04-07

**Authors:** Inna Šteinbuka, Aldis Austers, Oļegs Barānovs, Normunds Malnačs

**Affiliations:** ^1^Productivity Research Institute, University of Latvia, Riga, Latvia; ^2^European Policy Research Institute, Latvian Academy of Sciences, Riga, Latvia

**Keywords:** COVID-19 crisis, economic recovery, productivity, health care, public perceptions

## Abstract

The decision of EU and the response of the national governments to COVID-19 crisis provide the basis for returning “back to normal”. A key challenge is the transition to economic recovery in the presence of the ongoing COVID-19 risk. Adequate policy mix and forward-looking actions of the public institutions are crucial to mitigate the devastating impact of the crisis and to preserve growth. Governments need to facilitate positive changes in the labor market, adjust the macroeconomic and fiscal regimes, and mitigate the post-crisis “fatigue” of societies. The turmoil of the EU economy is symmetrical, as the pandemic has affected all EU Member States, but the impact of the pandemic varies considerably from one country to another, as does their ability to absorb the economic crisis. Also, variation in the vaccination performance is partly due to different institutional characteristics across countries. Small countries are more vulnerable to external economic shocks; however, they can increase their resilience by efficient governance and social response. Extraordinary pandemic crisis can be seen as a stress test for the small and open Latvian economy, and it is worth analyzing the lessons that Latvia had learned and its future prospects. The aim of this paper is to evaluate the economic and social consequences of the ongoing crisis in Latvia, assess the effectiveness of the response of the government to the crisis, analyse people's perceptions, and to identify the future scenarios. The authors applied a special theoretical framework for the assessment of the effectiveness of institutions. Institutional analysis of crises response by the Latvian government reveals that the government managed to avoid serious functional disruptions; however, it failed to show convincing ability to learn by doing. The authors also provide a comprehensive analysis of the macroeconomic trends of the “COVID-sick” Latvian economy and conclude that future-oriented solutions relate to international competitiveness and that the key factor of competitiveness is a productivity renaissance. The pandemic crisis has fostered the state support for healthcare, which in Latvia for decades has been underfinanced. The right choice of fiscal instruments is crucial to accelerate the economic recovery and better healthcare. Research is based on the macroeconomic assessment and survey-based analysis. The comparison of statistically justified findings with the public perception helps formulate conclusions on the future scenarios and policies.

## Introduction

The past 2 years have been powerfully shaped by the COVID-19 pandemic, and it is getting clear that the pandemic with its devastating effects will continue at least until 2022. Although successful vaccination programmes across advanced EU economies and high vaccination rate in Latvia provide the basis for returning “back to normal,” a normalization in 2022 should not be expected. Currently, less severe but very contagious Omicron has been spreading rapidly. The future prospects of endemic COVID-19, with new variants likely, means some ongoing domestic and border restrictions, repeating disruptions of supply chains, downturn in COVID-vulnerable economic sectors, negative labor markets' development, even if the economic costs will be less severe than in 2020 and 2021. In addition, one of the biggest global economic uncertainties is the trajectory for inflation, the associated policy responses, and its impact on post-COVID recovery.

A key challenge is a smart transition to economic normalization in the presence of the ongoing COVID-19 risk. What political response and forward-looking action are needed at this recovery stage to mitigate the devastating economic and social impact of crisis and preserve the sustainable growth? Governments and entrepreneurs need to respond to structural changes in labor market, to adjust the macroeconomic and fiscal regimes, and to find adequate policy mix in mitigating the postcrisis “fatigue” of societies. The aim of this paper is to assess Latvia's response to crisis from very different multidisciplinary angles.

In the *first* part, the focus of the research is on the institutional response and the interactions of the state and society during the crisis. As the pandemic crisis is a powerful stress test for the vulnerabilities and resilience of the governments, the authors tried to assess the effectiveness of the national institutions in crisis management. In our study, the Institutional Analysis and Development (IAD) (1) is used as a conceptual diagnostic tool for the assessment of the performance of the Latvian authorities during the pandemic. The harsh experience of the COVID-19 pandemic has split the societies. Latvians as well as the Europeans are divided over their belief in the national government's response and whether this response was effective. The people's perceptions mirror the effectiveness of the government's response to the pandemic. The findings are based on the Eurobarometer data, as well as on a specially conducted survey.

The *second* part focuses on the analysis of the macroeconomic consequences of the ongoing crisis in Latvia. The authors analyse the impact of the combined economic response of the EU and the national government to COVID-19 crisis on growth and the labor market. The COVID-19 crisis has aggravated the preexisting vulnerabilities and imbalances, and the lockdowns have amplified the socio-economic and regional inequalities across Europe. In Latvia, the faster convergence with the EU income level would be possible only if the Latvian economic growth would significantly overtake the EU average. Therefore, one of the major future challenges for the Latvian government is to find a proper policy mix to accelerate growth. Over a longer term, the key factor for growth based on strong competitiveness is the productivity renaissance. An accelerated programme of investment in technology and capital, coupled with complementary investments in the human capital and innovation, could raise a rather sharp growth in labor productivity. The macroeconomic research findings are based on a comprehensive statistical analysis, research studies, and policy documents. It aims at integrating the available data from a broad range of international and domestic sources from the perspective of Latvia. To determine the impact of the redistribution of labor resources on the overall productivity dynamics in the Latvian economy, a shift share analysis method was used.

The *third* part highlights Latvia's healthcare challenges before and during the COVID-19 crisis. The pandemic crisis highlighted the problems of EU and the Member States in coordinating with the healthcare policies. In Latvia, the crisis exerts enormous pressure on the health system, which was underfinanced for decades. On the other hand, the crisis had also positive implications on healthcare, primarily through increasing the wages of the medical staff and additional investments. The future scenario of healthcare development in Latvia is rather uncertain as the envisaged fiscal constraints in the years to come can overshadow the COVID-19 lessons, and lead to reconsidering the spending priorities in favor of other sectors. The study is based on the analysis of policy documents and statistical data from various data bases.

The study was supported by the National research programme, “Latvian heritage and future challenges for the sustainability of the state” for the project, “Challenges for the Latvian state and society and the solutions in international context” (INTERFRAME-LV, Project No.VPP-IZM-2018/1-0005).

## Latvia's Response to COVID-19 Crisis

The turmoil in the EU economy is symmetrical, as the pandemic has affected all the EU Member States, but the impact of the pandemic varies considerably from one country to another, as does their ability to absorb and respond to the economic crisis. Also, variation in vaccination performance is partly due to different institutional and structural characteristics across the countries. Small countries are naturally more vulnerable to external economic shocks; however, they can increase their resilience through the efficient response of the governmental authorities, businesses, and public. Extraordinary pandemic crisis can be seen as a stress test for the vulnerability and resilience of the small and open Latvian economy.

The analysis of Latvia's response to COVID-19 crisis has two dimensions: assessment of institutional response and assessment of state–society interaction during the crisis.

### Institutional Response

**Figure 1:**
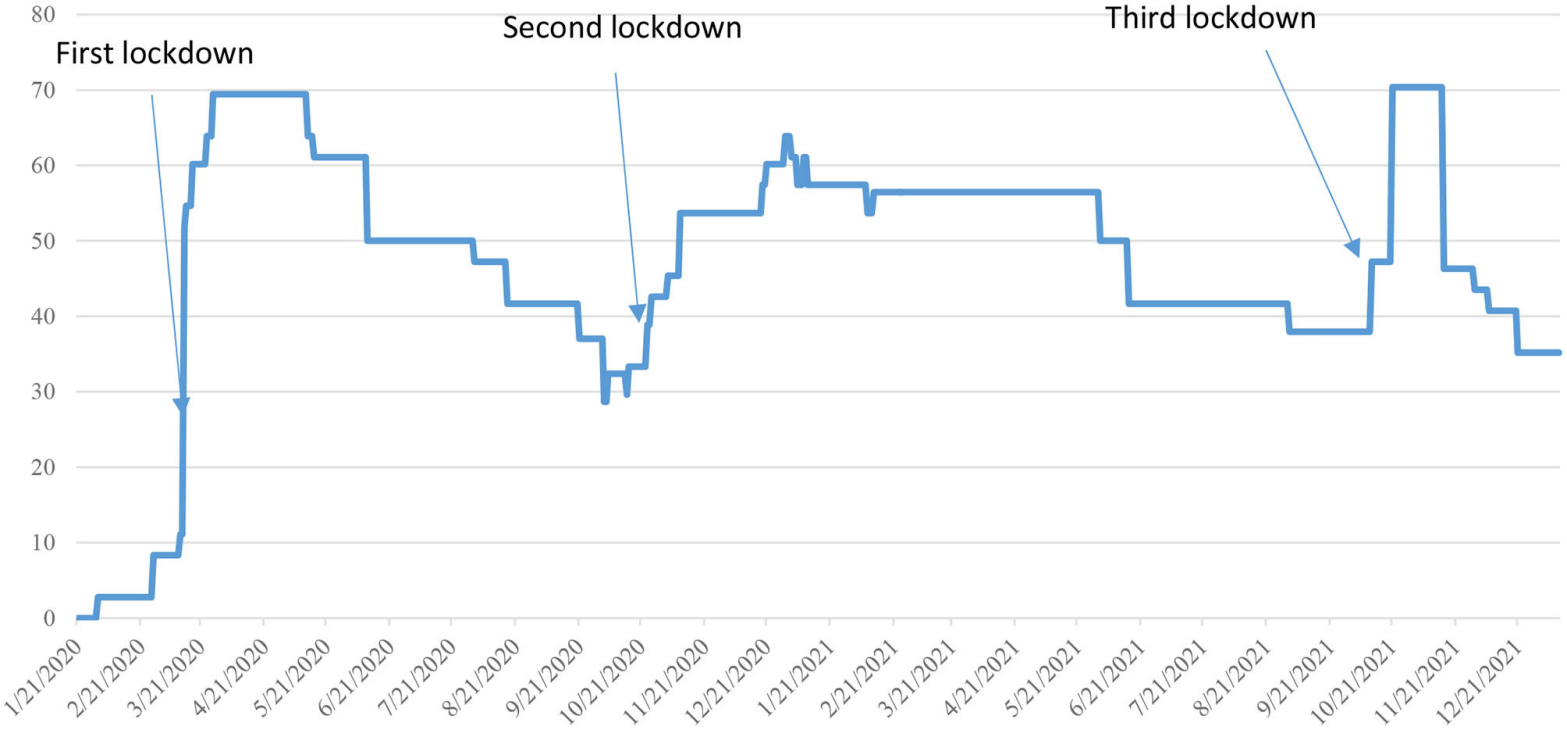
Government stringency index, Latvia, 21/01/2020-21/12/2021 (2).

Since the beginning of the COVID-19 pandemic in late 2019, Latvia has been through three major lockdowns, as shown in Figure 1 (2).

A study of institutional responses to the pandemic by the World Bank [(3), p.10] concludes that the scale and the unpredictability of the COVID-19 pandemic required the government systems that are “flexible, agile and limber,” “to steepen their learning curve” and to “quickly come up with contingency measures”.

In our study, the IAD is used as a conceptual diagnostic tool (1) for the assessment of the performance of the Latvian authorities during the pandemic. First, the IAD provides a comprehensive schema for tracking the essential processes inside any institution. Second, the IAD addresses the interactions between formal rules (i.e., legislative requirements that embody public morality) and informal rules (i.e., unwritten norms of behavior that embody civic morality) in shaping the institutional outcomes: the larger the gap between the formal and informal rules, the more is the space for illegal activity (4). Finally, a relevant feature of the IAD captures the transformative nature of the institutions: the more effective is the evaluation of the outcomes, the more effective and targeted are the learning and the adaptation of the participants over each institutional cycle.

**Figure 2:**
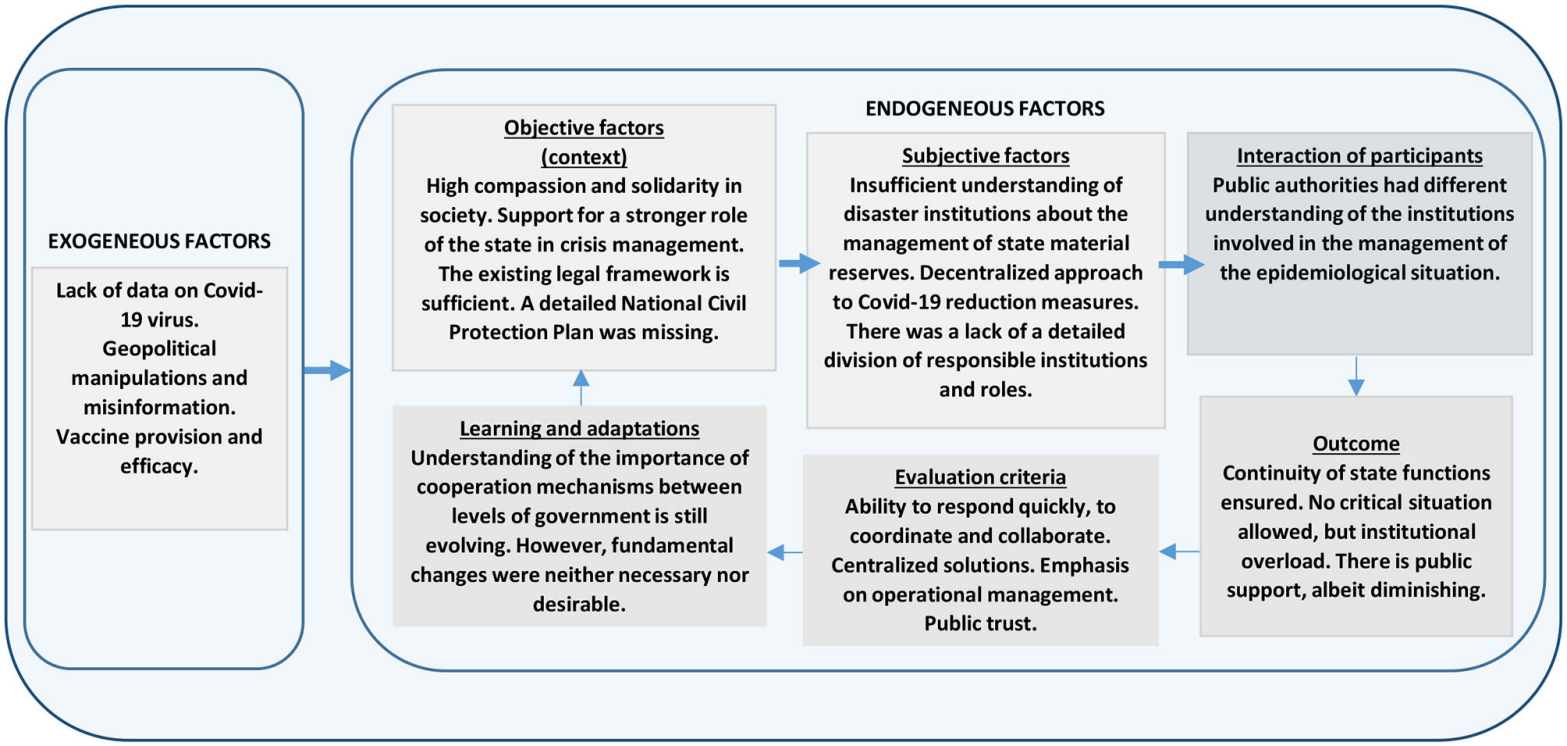
The assessment of the effectiveness of the work of Latvian public administration during the COVID-19 pandemic crisis (5). Source: Authors' analysis based on IAD framework.

Figure 2 (5) summarizes the insights on the performance of the Latvian authorities during the initial stages of the pandemic according to the IAD.

1) *Exogenous factors*. Like many other governments, the Latvian authorities were also faced with the lack of understanding the nature of the virus and the shortage of the data on the speed and scale regarding the spread of the infections. Also, access to vaccines and the efficacy of vaccines against the new strains of COVID-19 virus were another factor outside of Latvia's government's control. Yet another exogenous factor in Latvia's case was the impact of the “infodemic” influenced by geopolitical tensions and the spread of disinformation by Russia.
2) *Evaluation criteria*. The proposed criteria for the evaluation are based on the insights from the report by The State Audit Office of Latvia (6). These include: [1] the ability of the institutions to react quickly, to intensely coordinate with their activities and to cooperate with each other; [2] effective governance at all the levels of government, both central and local, thus fostering the public confidence in public administration; [3] centralized crisis management solutions, strengthening the role of central government; [4] effective operational coordination by monitoring the action plans and the achievement of the specified objectives; and [5] maintenance of public trust in state authorities.
3) *Objective (contextual) factors*. As to the formal rules, Latvia faced the pandemic without a valid National Civil Protection Plan and an effective disaster risk management system. Moreover, there was no constitutional regulation for emergency situations which created risks for legal proceedings in connection with the restriction of fundamental freedoms due to lockdowns. Nevertheless, neither the pandemic nor the emergency did result in such legal challenges that could have not been solved within the existing legal framework, although in certain areas (e.g., in relation to distance work), improvements in the regulatory environment were required. As regards the informal rules, an opinion poll revealed that after the first wave of the pandemic (September 2020), there was a high level of compassion and solidarity in the society, especially for the most vulnerable. The public advocated a greater role for the state in dealing with the crises caused by the pandemic, and in the event of a recurrence of the COVID-19 pandemic, would support equally strict or even stricter restrictions on people-to-2people contacts, even at the cost of such fundamental values of a liberal state as individual freedom and inviolability of personal life.
4) *Subjective factors*. At the beginning of the pandemic, the institutions responsible for disaster management did not have sufficient understanding of the principles of planning the state material reserves. Neither were there any detailed prescriptions for disaster management measures nor a list of responsible personnel. The planning and implementation of the COVID-19 mitigation measures were decentralized, as each municipality and state institution were in charge of its own resource planning and adequate supplies in the process of the provision for basic services.
5) *Interactions of participants*. In the absence of detailed criteria for the determination of which institutions are to be contacted in relation to the crisis management, each authority had developed its own understanding of the institutions involved in the management of the epidemiological situation. At the same time, the state institutions were able to ensure an uninterrupted continuity of state functioning, and a legally sound crisis management, even in the absence of constitutional regulation for emergencies.
6) *Outcome*. The restrictive measures implemented by the Latvian government during the first wave of the pandemic (March 2020) were sufficiently effective and were positively evaluated by the public. Critical situations with the provision of personal protective equipment were not permitted; however, the lack of information and common understanding of institutions in charge created a significant additional workload for the authorities.
7) *Learning and adaptations*. According to the State Audit Office, in Latvia, the understanding of the importance of adequate cooperation between the political leadership, public administration, and other institutions in charge of containing the spread of COVID-19 was still developing (6). While the public authorities were able to ensure the continuity of the public functions and prevent critical situations during the first pandemic in March 2020, the authorities were not able to demonstrate a strong and decisive leadership role in managing the second wave crisis of COVID-19 in Autumn 2020 and to prevent the third wave of massive infections in Autumn 2021. The second wave of the pandemic, in fact, surprised the government unprepared; it had not learned from the mistakes of the first wave; there was still a lack of an effective civil protection plan and of a common understanding of the principles of the epidemiological management across the public administration.

Many causes of the governance problems, a weak coordination of activities between the public institutions and economic sectors being the key challenge, as was a lack of focus and a virtually nonexistent assessment of the regulatory effectiveness and learning from identified policy shortcomings, were inherited from the prepandemic period (7). While the pandemic spectacularly exposed the problems in the public governance, it also presented an opportunity to break from the bad institutional inertia and to close the existing deficiencies. Lessons from the global experience demonstrated that high-level leadership and well-structured incentives, along with a degree of flexibility and focus on strategic issues were the preconditions for success. At the same time, countries were advised to refrain from complex designs, adding new structures to the overlapping functions, and eager for institutional borrowing from other contexts [(3), p.18].

### Assessment of State–Society Interaction

The study by the World Bank points out that along with the essential functions attributable to any effective governmental crisis response and contextual factors, such as the country's size and government capacity, variables, such as the quality of leadership, legitimacy, and trust in the government matter a lot, creating a feedback loop between the authorities and the public. On the one hand, it is argued that trust in the government may help to overcome the collective action problem and mount more rigorous response to the pandemic. On the other hand, the pandemic may also be an opportunity to build trust in low-trust environments, or, on the contrary, if the pandemic response falters, trust in the government may swiftly decline with a little chance of rebound [(3), p. 36–37].

A look at the state–society relations is particularly warranted in the case of Latvia, as Latvia is among those countries with a sizable shadow economy (8). The existence of a large shadow economy is an indicator not only of weak public institutions but also of a discrepancy between the public morality and civil morality.

**Figure 3:**
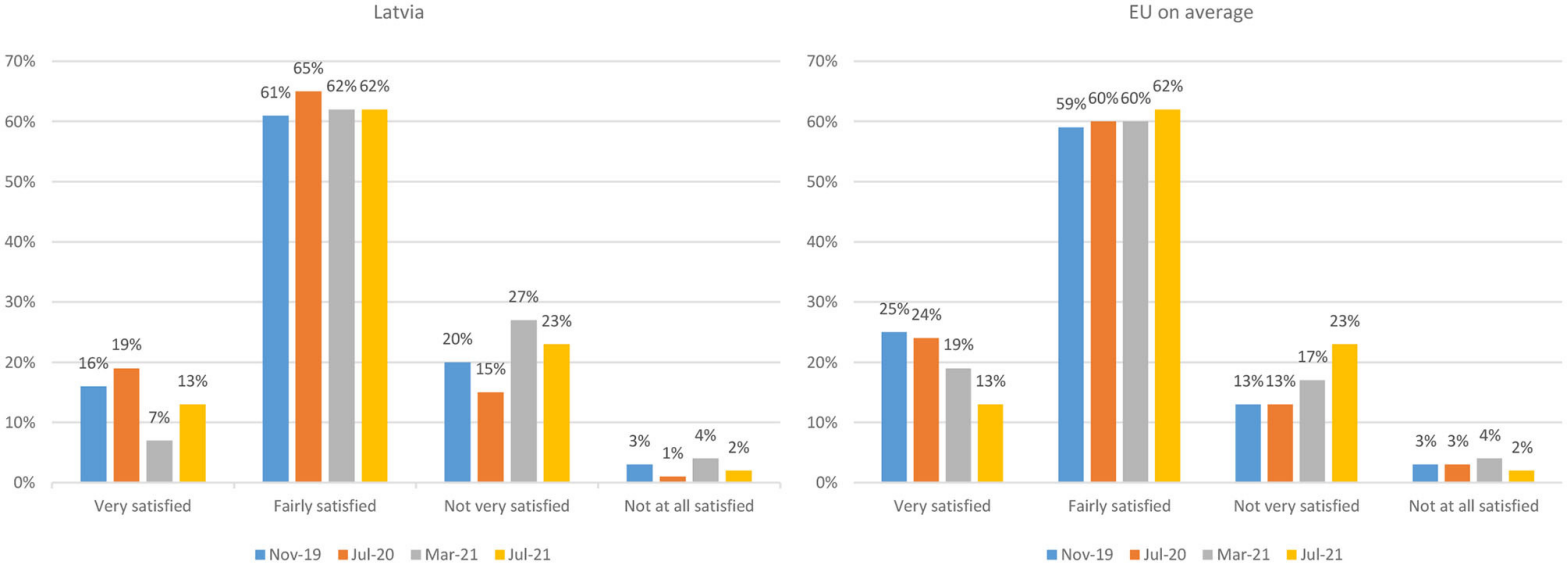
Personal satisfaction with the life people lead, percent of respondents, 2019-2021 (9). Source: Standard Eurobarometer 92.3, 93.1, 94.3, 95.3.

The Eurobarometer public opinion polls (Figure 3) (9) reveal that people's satisfaction with life in Latvia, albeit lower than in the EU on an average in general, during the pandemic, has been more volatile than in the EU on an average in which case one can notice a steady decline.

**Figure 4:**
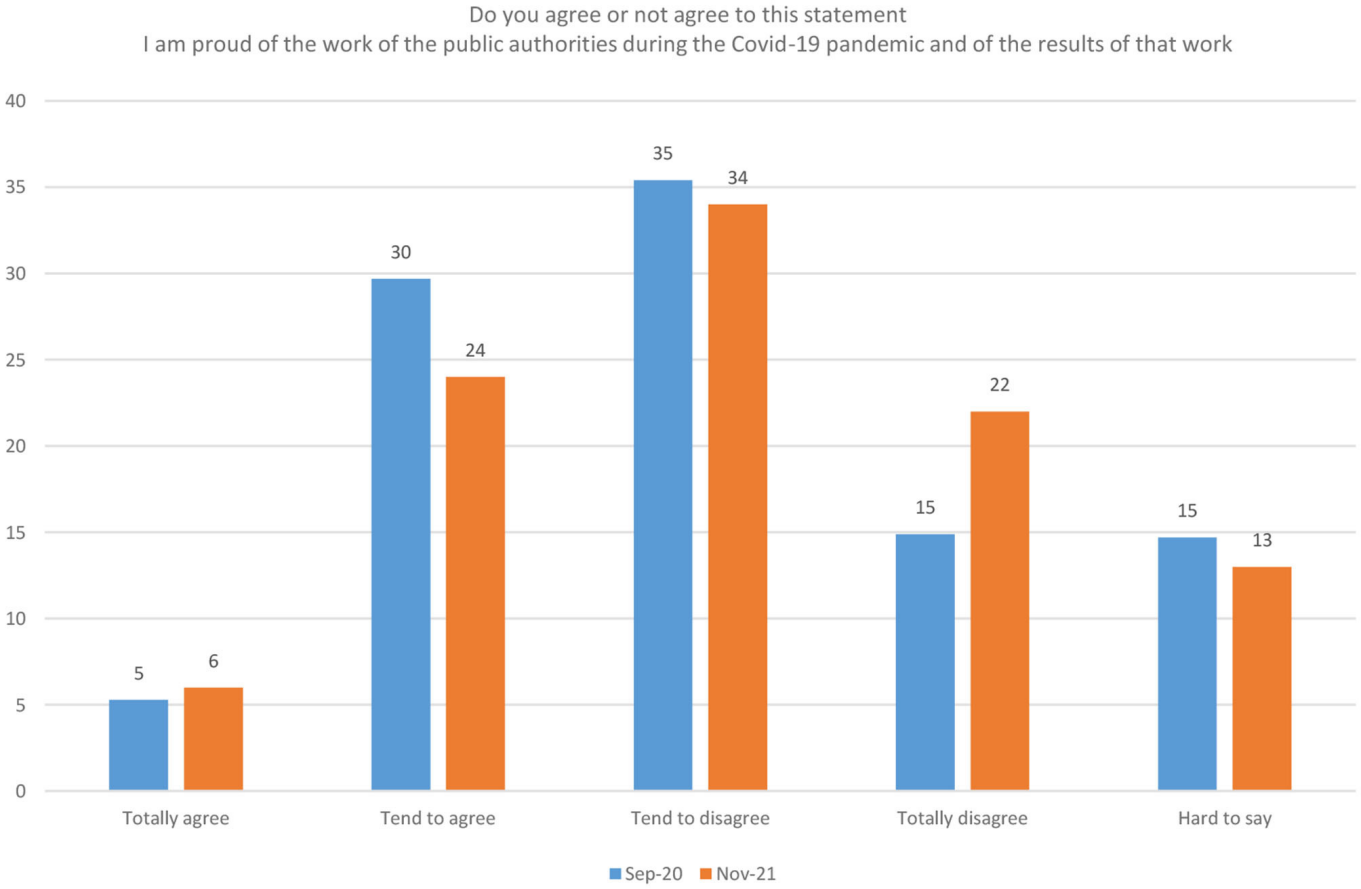
Public satisfaction with the work of public authorities during the pandemic, percent of respondents, 2020-2021 (10). Source: public poll carried out in the framework of this study.

However, a public poll carried out in the framework of this study (Figure 4) (10), showed that the Latvian public has been pretty in accepting the work of the public authorities during the pandemic. This acceptance declined during the later stages of the pandemic; nevertheless, it stayed relatively high considering the lack of foresight and decisive and consequent actions from the government.

**Figure 5:**
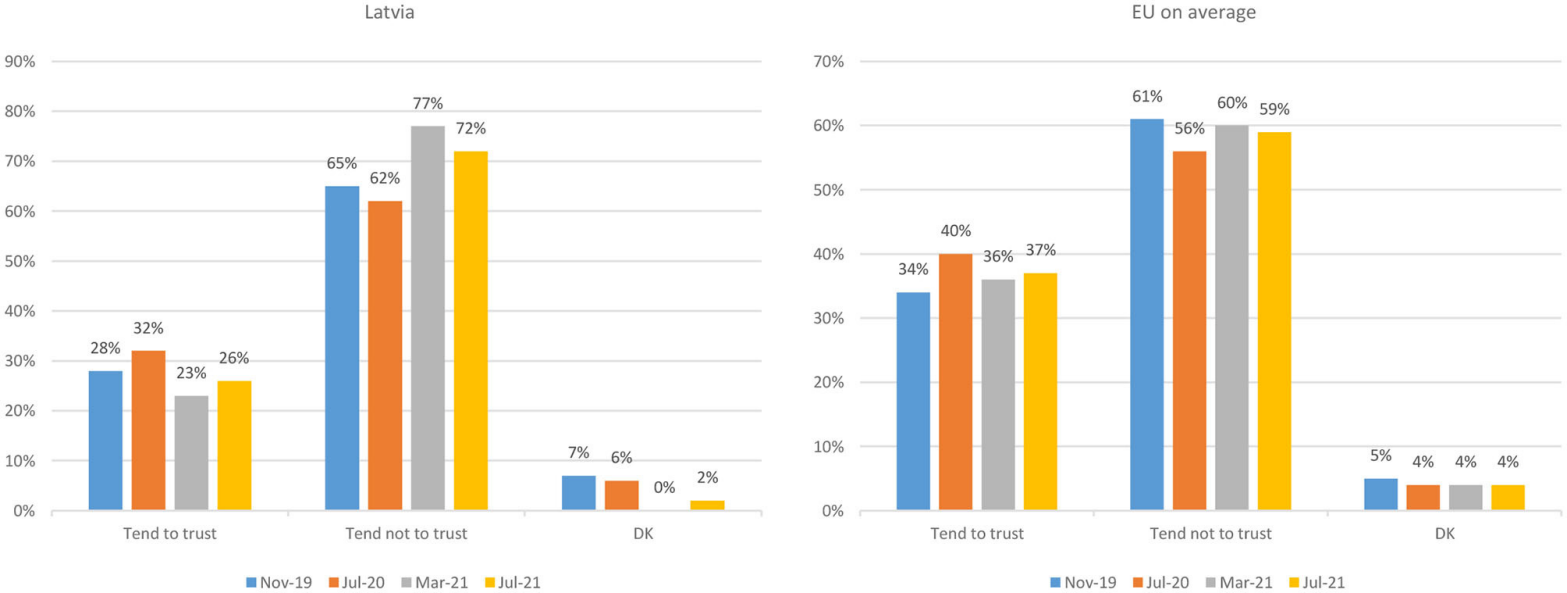
Public trust in national government, percent of respondents, percent of respondents, 2019-2021 (9). Source: Standard Eurobarometer 92.3, 93.1, 94.3, 95.3.

As to the trust in the government (Figure 5) (9), during the first wave of the pandemic, the public trust in the national government increased in Latvia which can be explained for the relatively low infection rates and less stringent lockdown measures adopted at that period. This surge in trust was followed by a sharp decline during the second wave of the pandemic which faced the Latvian government unprepared. In mid-2021, the trust began to recover; however, it is still to be determined how government's clumsiness with vaccination and the third wave of infections in Autumn 2021 has affected the trust. This allows us to conclude that in the case of Latvia, the public trust in the government has been tracking the perceived quality of governmental response: although initially the pandemic provided an opportunity to build trust in the low-trust environment, at later stages, when the pandemic response faltered, the trust in government swiftly fell.

### Vaccination Against COVID-19—Another Stress Test

Vaccination against COVID-19 presented another stress test to the effectiveness of governmental policies and state–society relations. Although the first significant doses of vaccines reached Latvia in January 2020, mass vaccinations started only in the second part of April. A brief period of catch-up of vaccination in Latvia was followed by a slowdown in May and June, which lasted until early October. On October 8, the government of Latvia, faced with a rapidly growing number of cases of infection and overburdened hospitals, was forced to declare yet another public emergency, to reintroduce strict measures of social distancing, and to considerably widen the list of those professionals for whom the vaccination is mandatory. Only after October, Latvia was able to catch-up significantly in vaccination rates with its neighboring countries, and even by-pass several of them like Poland and Estonia (11).

The vaccination process in Latvia had been punctuated by several public scandals in 2021. At first, an outcry was caused by the failure of the authorities to procure enough vaccine doses in the face of the failure of AstraZeneca to obtain an early authorization for use in the public from the European Medicines Agency. The initial vaccination strategy of the Latvian authorities had been based on AstraZeneca vaccine, which was cheaper and easier to handle with. Later, during the shortage period of vaccines, the approach of the government for the selection of priority groups was questioned. Upon the arrival of enough vaccines to start a mass vaccination, the management of the vaccination campaign became a target of public criticism. During the second part of 2021, the authorities were faced with an increasing public resistance to allegedly “compulsory” measures of vaccination. What is more, the Ministry of Health created a new body under the direct supervision of the ministers with the purpose to ensure a better coordination of the vaccination process; however, soon after its establishment, the activities of this Vaccination Bureau led to the role of conflicts with the already existing institutions like the National Health Service and on numerous occasions, confused the patients over vaccination order and timing.

However, as argued by a comparative study on the societal response to the stringent governmental measures aimed at stemming the spread of COVID-19 infection in 2020 (12), not only the quality and form of governmental action, but also the cultural traits and the form of government matters. So, the collectivist and democratic countries have been able to implement relatively more effective measures aimed at constraining the geographic mobility of the people, compared to more individualistic and authoritarian countries. It is argued that as people in democracies are endowed with more social capital and trust in cooperation, they are more likely to follow and support government interventions. At the same time, while individualistic societies tend to be more dynamic and innovative, this individualism makes the collective coordinated response to a pandemic harder.

Indeed, the success in containing the spread of the virus does not depend solely on the actions of the government, but on the society as well. While during the early phases of the pandemic this societal role was confined to a more passive kind of measures like social distancing, abstention from travel etc., then during the vaccinations, people were expected to commit themselves to a more active role, to accept injections of vaccines, at a risk of personal discomfort or health damage.

**Figure 6:**
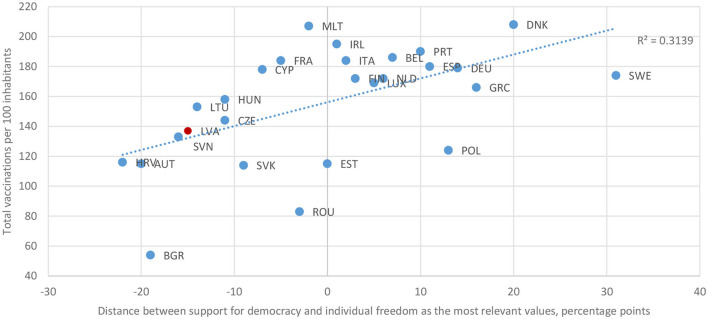
Correlation between people's attitude toward democracy* and the speed of vaccination** in EU member states (13, 14). Notes: (*) The ranking of EU member states is based on the difference between the share of those opinion poll respondents who chose “democracy” and those who chose “individual freedom” as the most important value for them personally: the more negative the difference, the bigger the share of respondents preferring individual freedom over democracy. (**) Vaccination data on 30 December 2021.

Analysis of data from the Eurobarometer opinion poll confirms the assertion that democratic- oriented societies in the EU tend to advance in vaccinating people somewhat quicker than the societies where individualism prevails. The outcome of this analysis is shown in Figure 6 (13, 14). It follows that probably more widespread vaccine mandates should be considered by governments in countries with a more individualistic value system.

The data from an opinion poll in Latvia (15) reveal some intriguing correlations regarding the vaccinated and not vaccinated people in Latvia. Thus, those people who consider themselves to be in a risky group of people for whom COVID-19 can cause serious health problems or even be fatal tend to be more attentive to vaccination (Table 1) (15). Also, the people whose family members or close friends have been heavily ill or even died from the COVID-19 are more likely to have vaccinated themselves against COVID-19.

**Table 1:** Vaccination status of people in the context of their exposure to the COVID-19 illness (15).

		**Do you belong to the group of people for whom the COVID-19 infection may result is severe consequences or even be fatal?**			**Have you or anyone from your family or close friends been seriously ill with COVID-19 or even died?**		
		**No**	**Yes**	**Total**	**No**	**Yes**	**Total**
		**Pearson chi**^**2**^**(1)** **=** **11.8316 Pr** **=** **0.001**			**Pearson chi**^**2**^**(1)** **=** **26.2663 Pr** **=** **0.000**		
Have you ever been vaccinated against COVID-19 at least once?	No	*n* = 215	*n* = 59	*n* = 274	*n* = 223	*n* = 81	*n* = 304
		78%	22%	100%	73%	27%	100%
		**34%**	**22%**	31%	**37%**	**22%**	31%
	Yes	*n* = 416	*n* = 204	*n* = 620	*n* = 373	*n* = 292	*n* = 665
		67%	33%	100%	56%	44%	100%
		**66%**	**78%**	69%	**63%**	**78%**	69%
	Total	*n* = 631	*n* = 263	*n* = 894	*n* = 596	*n* = 373	*n* = 969
		71%	29%	100%	62%	38%	100%
		100%	100%	100%	100%	100%	100%

Those people who have been fully or partially vaccinated strongly tend to agree with the view that the vaccination against COVID-19 must be treated as an obligation to one's own health and to the health of others. In the meantime, the vaccinated people tend to disagree with the opinion that mandatory vaccination should not be supported, although this correlation is not very strong (Table 2) (15).

**Table 2:** Correlations between people's views on vaccination obligation (15).

	**Opinion**	**1**	**2**	**3**
1	Have you ever been vaccinated against COVID-19 at least once?	1		
		*n* = 987		
2	Vaccination against COVID-19 is a duty for your own health and that of others	0.4437	1	
		0.000000		
		*n* = 905	*n* = 916	
3	Mandatory vaccination against COVID-19 should not be supported	−0.3418	−0.5114	1
		0.000000	0.000000	
		*n* = 892	*n* = 845	*n* = 904

Finally, the factor analysis of the respondent's responses to the statements included in the opinion poll was best combined in three opinion groups (Table 3) (15):

**Table 3:** The dominant opinion groups in Latvia in relation to the vaccination against COVID-19 (15).

***N* = 463**	**Skeptics**	**Semsible citizens**	**Enlightend warriors against the COVID-19**
	**Comp 1**	**Comp 2**	**Comp 3**
Countries where people trust and support each other develop faster		0.6721	
Tax evasion is reprehensible		0.4799	
Vaccination against COVID-19 is an obligation for your own health and that of others	−0.4241		
Vaccination poses greater risk to health than COVID-19	0.4512		
Vaccination of the Latvian population against COVID-19 has a positive effect on Latvia's economic development	−0.3846		
The COVID-19 pandemic and related vaccination is a ploy staged by the pharmaceutical industry to enrich itself	0.445		
I am proud of the work of the Latvian authorities during the COVID-19 pandemic and of the results of this work			0.5102
Mandatory vaccination against COVID-19 must not be supported	0.4589		
In Latvia, vaccines against COVID-19 are available to anyone who wants them		0.5268	
The fight against pandemics such as COVID-19 is best coordinated not at national level but at European Union level.			0.7569

1) *Skepticism as a worldview*. People who have not vaccinated tend to express negative views not only about vaccines and vaccination, but also about the importance of vaccination for the development of the state. They do not show much trust in the public authorities, have low tax morale, and have similar sociodemographic profile, most of them are middle aged (45–63) males with primary education, employed in the private sector or not working at all, with low incomes and living outside of large urbanized areas.
2) *Sensible citizens*. People in this opinion group share the view about the positive impact of vaccination on national development, condemn tax evasion, and believe that vaccines are available to everyone in Latvia.
3) *Enlightened warriors against COVID-19*. In this opinion group, people share the proudness of the work by the Latvian authorities during the pandemic and would like to push for more action at the European level.

## Macroeconomic Consequences of the COVID-19 Crisis

This section presents the overall impact of the COVID-19 pandemic on the macroeconomic development of the EU, which has been considerably softened by the rapid economic response of EU. The authors analyse whether the major growth and jobs trends of the “Covid-sick” Latvian economy correspond with those in the EU. Another important angle of the study are socio-economic and regional inequalities across Europe and in Latvia. Clearly, the crisis is slowing down Latvia's economic development and the improvement of the living standards of the population. The core issue is on how to accelerate the growth. Low productivity is not a facilitating breakthrough, and therefore the research is focused on the policy actions to boost the productivity and accelerate the growth.

### The Impact of the COVID-19 Pandemic on the EU Economy and the EU Response

The COVID-19 pandemic resulted in an unprecedented economic contraction in 2020. Due to the pandemic and the ensuing containment measures, economic activity contracted in almost all the countries of the world with EU real GDP falling by 5.9% in 2020.

The *economic response* of EU to the crisis was forceful and, compared to previous crises, much faster. The support to the economy has been provided in three phases. The first *emergency steps* helped mobilize the EU resources, including €82 billion from the EU budget and national budgets. To accumulate public resources in the Member States for mitigating the economic damage caused by the COVID-19 pandemic, the EU deployed the General Escape Clause of the Stability and Growth Pact allowing the EU Member States to increase the general government deficit, as needed, till 2023. The EU has also activated the temporary framework for state aid allowing for immediate support to businesses. These measures allow the Member States to provide fiscal and liquidity support to their “Covid-sick” economies, which amounted to €3 trillion in 2020. The actions of the EU to support the financial stability were backed up by the European Central Bank.

The second, the *repair phase* used solidarity through the mobilization of EU instruments amounting to €540 billion to cushion the economic impact of the crisis, including the temporary Support to mitigate Unemployment Risks in an Emergency (SURE).

Finally, in the *recovery phase*, the €2 trillion firepower of the new Multi-annual Financial Framework and Next Generation EU, in particular, the historical recovery package, the Recovery and Resilience Facility (RRF), will support the recovery, while making the economies of EU and societies more resilient. Although the effectiveness of the implementation of these financial packages remains to be seen, the crisis has led to a fundamentally new approach to the EU economic support mechanism.

Thanks to the strong and well-coordinated EU crisis response, the damage to the EU economy appears considerably less than feared at the outset of the COVID-19 pandemic (17 ppxi).

As vaccination campaigns progressed and restrictions gradually lifted, economic growth has started to resume since Spring 2021. However, the unevenness of the recovery between EU Member States is widespread. Denmark and Sweden have all achieved very good results. Meanwhile, several European countries, such as Germany and Italy, have done worse. Spain had the hardest time during the pandemic.

**Figure 7:**
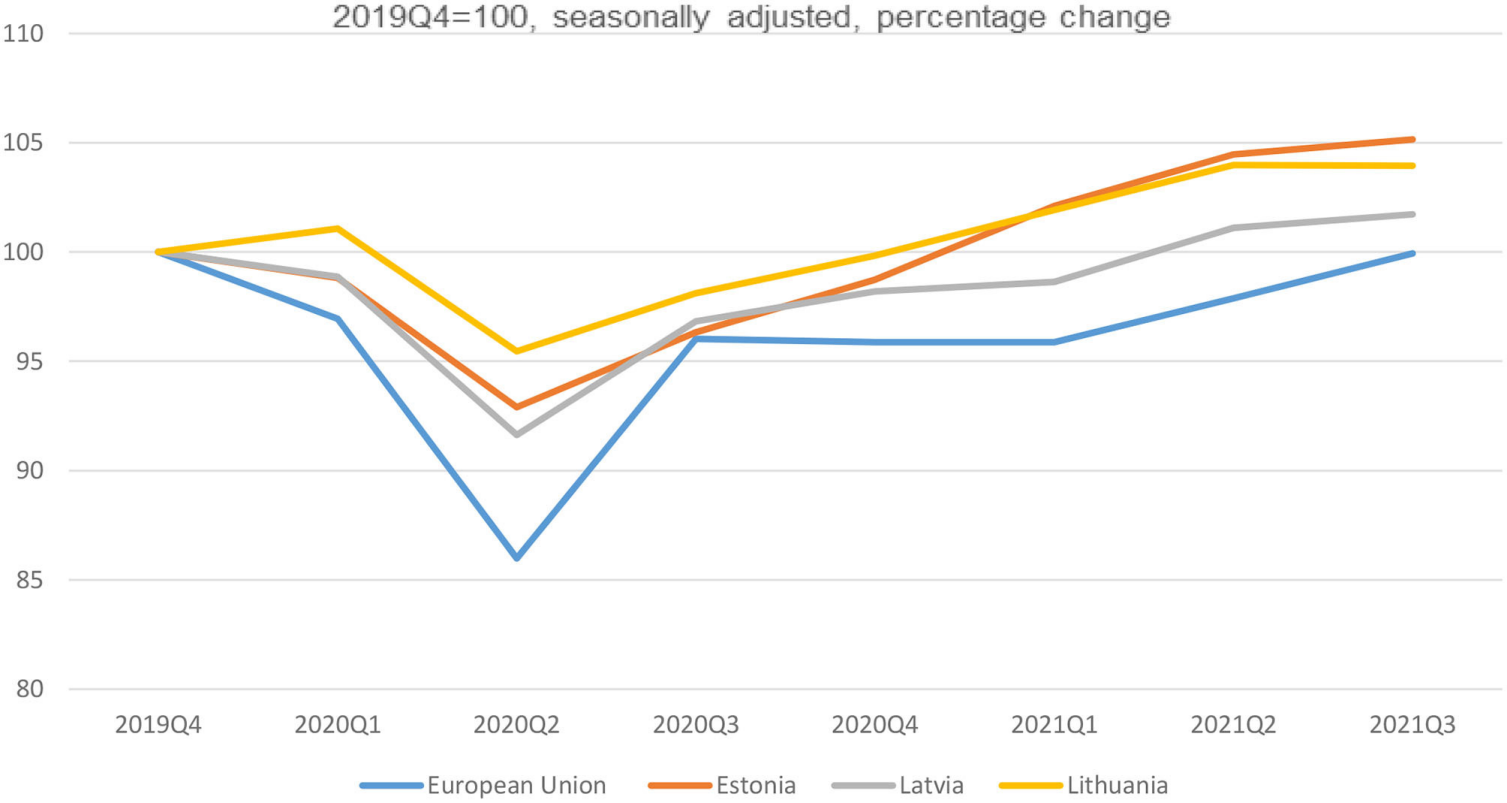
Growth rates of GDP in Baltic states and the EU (16). Source: author's construction based on the Eurostat data, 2021.

Output in most EU countries in 2021 surpassed its late-2019 level and is converging on its prepandemic plane. This is true also for the Baltic States where the situation in the terms of economic growth seems better than in the EU on an average (Figure 7) (16).

Despite the larger decline in the GDP, the labor market in the EU remained resilient as job retention schemes and other measures, such as the EU-level SURE instrument have protected the employment. However, the economic impact of the COVID-19 pandemic has been uneven across the population groups. Employment fell most among low-skilled workers, as they are more likely to accept jobs that require physical proximity and are less likely to be able to telework.

As the economy expands, the labor market is forecast to complete its recovery in 2022. An estimated 3.4 million jobs are projected to be created in 2022 and 2023, bringing the unemployment rate in the EU down to 6.5% in 2023 (17).

Although the impact of the pandemic on economic activity has weakened considerably in 2021, COVID-19 has not yet been defeated and further recovery is heavily dependent on pandemic evolution and vaccination coverage. Economic risks also relate to the potentially protracted impact of the current supply constraints and bottlenecks and rising inflation, driven largely by a spike in energy prices.

The COVID-19 crisis has aggravated the preexisting vulnerabilities and imbalances. For instance, lockdowns have clearly amplified the socio-economic and regional inequalities across Europe. Furthermore, the pandemic has added to the chronic pre-crisis “decreases” (population aging, weak productivity growth, income inequality, and territorial disparities within and among the Member States). Internal imbalances related to high government and private debt have increased. Prepandemic dynamic house price trends persisted and mortgage debt continued to grow significantly in some countries. Current account deficits widened in the countries dependent on tourism revenues. Addressing these challenges requires investment and structural reforms. New reform dynamics is rather visible in the areas of climate change and digital transformation. However, the key postcrisis challenge is linked to reducing high and divergent public debt ratios in a growth-friendly manner (18).

### Growth and Labor Market in Latvia in 2020–2021

Latvia's economy withstood the initial blow of the COVID-19 pandemic, which began in March 2020, relatively well. On a quarterly basis, the largest decline was observed in the second quarter of 2020, when the economy shrank by 8.9%. However, it was a relatively modest decline compared to the EU, where GDP contracted by 13.9% on average. Overall, in 2020, the GDP decreased by 3.6%, compared to 2019. The most significant reductions in 2020 were in the accommodation and food, beverage, and leisure industries. The restrictions imposed on COVID-19 also had a significant impact on aviation, land transport, and railway companies.

In 2021, economic activity became gradually increasing. In the second quarter of 2021, the GDP was 10.8% higher than a year ago, and the economy exceeded preCOVID-19 levels. In the third quarter, the growth rate reached 5.1%. The pickup in the growth was broadly based, led by the recovery of the pandemic withheld consumer demand, the growth of EU-financed public investment, and exports. The COVID-19 pandemic continued to affect the industries with a high share of social contact, while industries that are more export-oriented were developing more successfully, and strong positive trends were observed in the various sectors of the manufacturing industry.

Exports of goods play an important role in mitigating the negative effects of the COVID-19 pandemic. As supply chains are shortening, Latvia benefited from additional export opportunities and increased its competitiveness in the European commodity markets. Since most of the Latvian firms are small or medium-sized, it was relatively quicker and cheaper for them to adapt to the new circumstances than for the large companies. At the same time, exports of services lag far behind the precrisis levels.

The COVID-19 crisis has had a moderate impact on investment. Despite the overall decline in the economic activity in 2020, investment increased. Positive trends can also be observed in 2021. The government's policy of introducing the “Green Channel” principle (19) to priority investment projects facilitation of investment attraction. The net inflow of FDI attracted in Latvia in 2020 increased by 10.2% compared to 2019 and reached 3% of GDP. In the first 9 months of 2021, the net FDI inflows attracted to Latvia reached 5.6% of GDP. The increase in FDI was largely driven by reinvested earnings, which increased almost 2.5 times year-on-year. Investment is expected to increase further, supported by the improving economic sentiment and the significant inflow of the EU funds.

The imposition of restrictive measures in response to the epidemiological worsening had a significant impact on the labor market. Since mid-March 2020, the restrictive measures have hardly hit labor-intensive sectors. In total, the number of employees decreased by 1.9% or about 17 thousand in 2020, which is the largest reduction in the number of employees since 2010. Simultaneously, the unemployment rate in Latvia in 2020 reached 8.1%, exceeding the level of 2019 by 1.8%points.

State-support mechanisms, such as downtime support and wage subsidies have helped companies to maintain jobs and protect people from total loss of income. The support has also been provided to the vulnerable groups, including the unemployed.

Recent employment and unemployment figures show that the labor market has largely overcome the deepest point of the COVID−19 pandemic crisis and is gradually adapting to new circumstances. Since April 2021, unemployment continues to fall.

However, the crisis has left its mark on the labor market. The number of employees and the employment rate are still significantly lower than in 2019. In the 3 quarters of 2021, the number of persons employed was by almost 32 thousand or 4% less than in the corresponding period of 2020.

The crisis has badly affected the economic activity of the population. Weakening of economic activity in combination with the negative demographic trends of Latvia has led to the reduction in the labor supply and increased the risks of labor shortages. During the crisis, the share of long-term jobseekers has gradually increased, which, together with regional labor market disproportions, will trigger the risks of structural unemployment in the coming years, as well as exacerbate the problem of labor supply.

Overall, the labor market situation is expected to gradually improve, leading to more jobs and lower unemployment. However, it will become increasingly difficult to find qualified professionals in the situation of large regional labor market disparities, especially in such sectors as construction and manufacturing.

Despite the widespread impact of the COVID-19 crisis on the labor market, the overall wage dynamics remain on the upside, driven by both higher-skilled and better-paid jobs in the labor market and limited labor supply. The average monthly gross salary in the third quarter of 2021, compared to the corresponding period of the previous year, increased by 10.4% to an average of €1,280 per month, which has been the fastest growth of the average salary in Latvia in the last 13 years.

It should be noted that a significant increase in wages in Latvia has been observed already in the precrisis period. The average increase in wages over the last 5 years has been close to 7% per year. The process of wage convergence toward the economically developed countries of the EU and the growing shortage of skilled workers in the shrinking Latvian labor market foster the entrepreneurs to think not only on how to attract new specialists, but also on how to keep the existing ones. One could expect that the entrepreneurs will maintain their pressure on wage increase.

Although the uncertainty about the impact of COVID-19 on economic development is still extremely high, most experts predict that 4–5% growth in Latvia will continue in 2022 (20, 21).

The further development of the economy in the medium term depends on the external environment and the pace of reforms.

### How to Accelerate Growth?

The main indicator of Latvia's welfare, GDP per capita is the fourth lowest in the EU (after Bulgaria, Romania and Croatia). The faster convergence with the EU income level would be possible only if the Latvian economic growth would significantly overtake the EU average. Therefore, one of the major future challenges for the Latvian government is to find a proper policy mix to accelerate growth.

One of the reasons of slow growth compared to the highly developed countries of the EU is the low productivity. In 2020, productivity in Latvia was 51.7% (almost 71.9% in Purchasing Power Standards) of the EU average, which is one of the lowest indicators in the EU. This deviation from the EU average level is mainly explained by the low productivity of total factors, with significant differences in the quality of production resources (human and capital).

Maintaining productivity growth will not be simple, as easy gains have already been exhausted and firms are approaching their technology frontiers. Continued progress in implementing structural reforms will be needed to reduce the productivity gap, improving the governance of public enterprises, improving the business environment, modernizing the public infrastructure, and strengthening the judiciary (22).

There has been a growing recognition, that promoting proproductivity policies can be a particularly daunting task. When it comes to productivity, there is neither a silver-bullet solution, nor a standard set of reforms that can be implemented in the same way in every country [(23), p. 197].

The competitive advantages of the Latvian economy are mainly based on technological factors, improvement of production efficiency, innovation, and digitalisation. The crisis caused by the COVID-19 pandemic is a catalyst for more rapid change (digitalisation, teleworking, etc.). However, the fundamentals of productivity remain unchanged and relate to investment in human capital, investment and capital intensity, the ability to integrate into global value chains, and increase in the export potential, innovation, the development of new products, services and methods, and so on.

**Figure 8:**
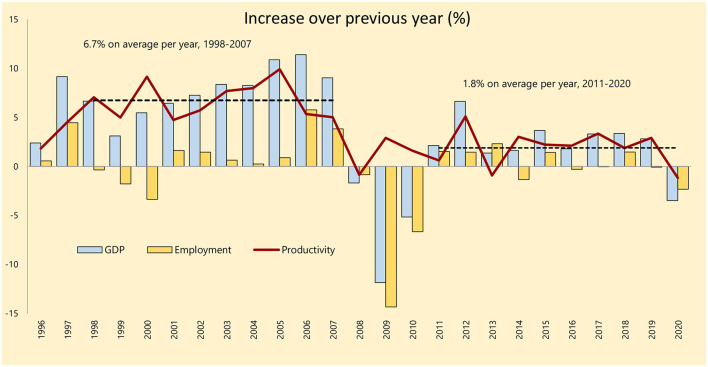
Annual productivity growth rates in Latvia (16). Source: author's construction based on the Eurostat data, 2021.

The slowdown in the productivity convergence in the last decade (Figure 8) (16) points to a “productivity trap,” which requires overcoming structural reforms and significantly improving the innovative solutions.

The low level of productivity in the Latvian economy is largely determined by the extremely low productivity in the manufacturing industry due to structural factors. As the experience of developed countries shows, it is the manufacturing industry that has a potentially higher innovation capacity. The structure of Latvia's manufacturing industry is strongly dominated by low-tech industries, which in 2020 account for more than half of the total value added to the manufacturing industry, which is almost one and a half times more than the EU average (16).

Technological factors, such as the modernization of production, the improvement of the existing technologies, and the introduction of new technologies, play a key role in raising the productivity levels. The transition from old to newer technologies contributes to the productivity growth at the company and industry level. However, the performance of such changes in raising the overall productivity levels depends to a large extent on the redistribution of resources from the lowest to the highest productivity sectors, as well as to sectors with faster productivity dynamics.

**Figure 9:**
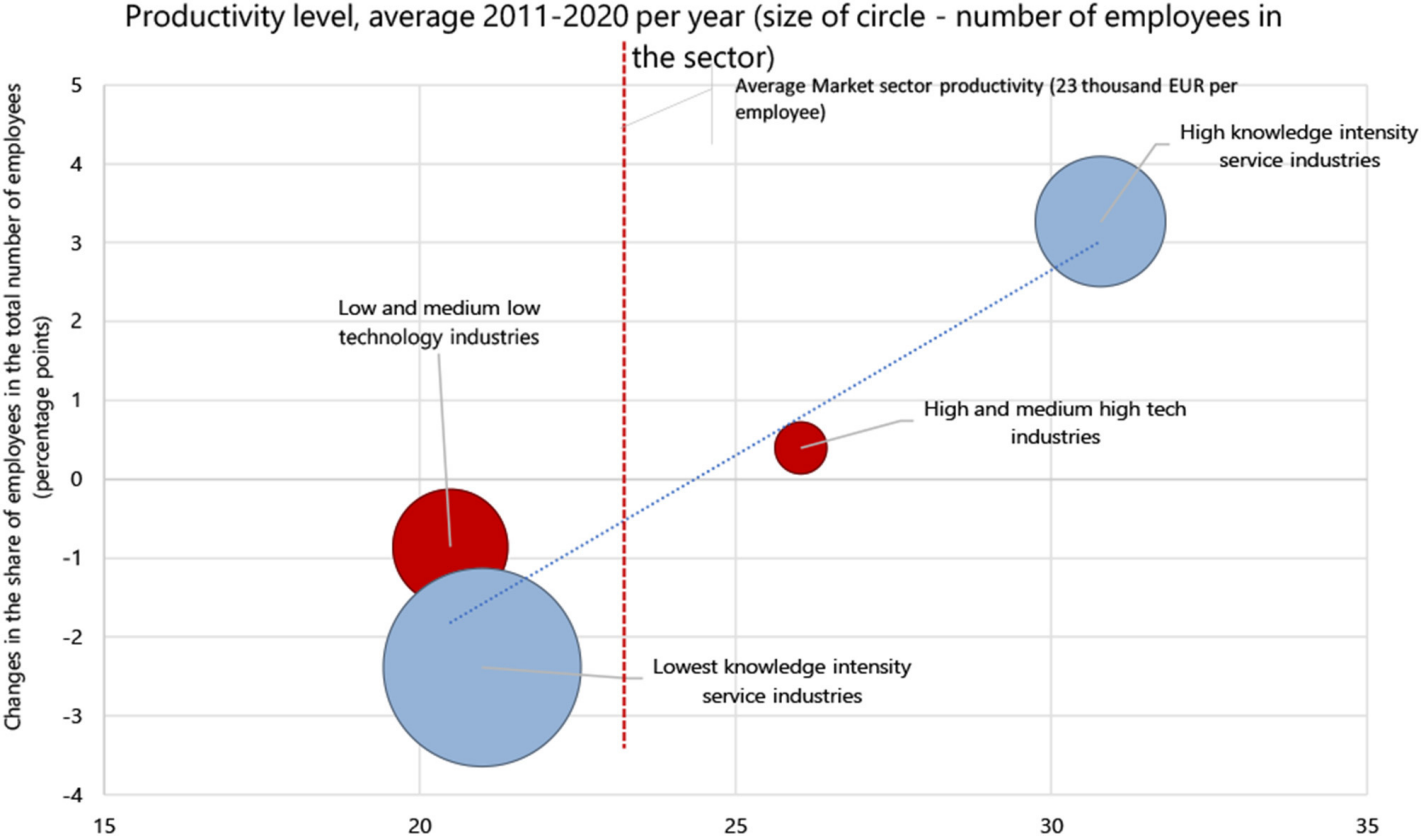
2011–2020 changes in the structure of employment in sector aggregated by technology levels with differing productivity levels (16). Source: author's construction based on the Eurostat data, 2021.

The shift shares analysis method used shows such that employment keeps growing in sectors with above-average productivity, such as computer and electronic equipment, while employment in some low-productivity sectors, such as light industry, keeps declining. However, job creation is still high in sectors with relatively lower productivity levels, such as accommodation and food service activities. In general, the redistribution of labor resources in favor of productive sectors is insufficient to have a significant impact on the faster growth of the overall productivity in the economy (Figure 9) (16).

Productivity growth will have to increasingly rely on knowledge-intensive activities. Latvia's weakest point has been innovation, which requires investments in research and development, in developing people's knowledge and skills, and in other intangible assets [(24), p. 4].

In the 2021 European Innovation Scoreboard published annually by the European Commission, Latvia ranks 25th among the 27 EU countries and is included in the group of the Emerging Innovators (25). Latvia's low innovative capacity is not favorable for the future and is currently significantly limited by the low quality of research institutions, weak international cooperation in science and research, weak cooperation between scientists and entrepreneurs, low level of investment in research and development, and other factors.

Digitization is another determinant of productivity. Latvian companies lag significantly behind in the use of digital technologies, entrepreneurs lack digital skills and knowledge, skills, and appropriate tools (for example, productivity tools for digital trade, cross-border online trade, etc.) compared to OECD Member States.

Although after the introduction of high-speed broadband network, Latvia exceeds the OECD and EU average level; only a few Latvian companies use new digital technologies, such as the analysis of large databases, radio frequency identification technology, etc.

In the ranking of 2021 Digital Economy and Society Index (DESI), Latvia ranks 17th (26) among 27 countries. In Latvia, a digital divide has come about between the city and countryside. Much of Latvia's population lacks the digital skills needed to make the effective use of the internet. The integration of digital technologies in businesses is well-below the EU average. Basically, Latvia's population is not fully prepared for a digital boom in the economy. Latvia has one of the highest proportions of inhabitants in various age groups with low overall levels of digital skills. This not only leads to a shortage of digital skills on the labor market, but also generally hinders the broader rollout of digital technologies within the companies. Core policies should increase the digital skills for society as a whole, with a specific focus on each target group, to avoid the risk of future imbalances. An overarching strategy for the digitalisation of business should be drawn up. An important tool in the digital age is the ongoing dialogue with businesses about the development of new technologies and the impact of trends in the digital economy on the way these work.

The prospects for productivity growth in the future are closely related to the deeper integration of Latvian companies into global markets, increasing the share of knowledge-intensive products and services in total exports.

Latvia's relatively low level of exports and attracted FDI compared to the rest of the Baltic States and the EU averages indicates insufficient participation in the global production and supply chains. The OECD's 2017 Economic Report on Latvia also notes that a small proportion of companies participate in global supply chains. Companies participating in the global supply chains have higher productivity, employment, and wages on average [(27), p. 10].

The availability and quality of the workforce play a key role in increasing the productivity. The main directions for improving the availability and quality of the labor force that are relevant to Latvia are the following: addressing the demographic and migration issues, improving the availability and quality of education at all levels, and promoting retraining and further training.

To promote the development of human capital, several reforms have been implemented or started in Latvia, the positive impact of which on the overall level of productivity can be expected only in the medium or long term. The main challenges of the Latvian labor market in the medium term are related to the aging of the labor force and the shortage of labor. The aging of the workforce will have the greatest impact on the availability of medium-skilled labor, especially in sectors, such as transport and storage, construction, manufacturing, and agriculture and trade. Labor shortages may also occur in sectors where the demand for higher-skilled labor is expected to increase significantly, in the professional, scientific, and technical services and in information and communication services, in particular in the areas of STEM.

The Ministry of Economics of Latvia forecasts that in the medium and long term, if the current structure of labor force training is maintained, the following significant labor market disproportions are expected (28):

° lack of highly qualified specialists in natural sciences, ICT, and engineering.
° surplus of the workforce with higher qualifications in social sciences, business and humanities sciences.
° shortage of labor force with vocational secondary education.
° surplus of the workforce with secondary general education, basic education and lower levels of education.

As the problem of labor shortages in the labor market will intensify in the future, it is necessary to strengthen the adult education system to ensure the transition of the labor force from unproductive to growing sectors. The efficiency of the adult education system will also play an important role in mitigating the negative effects of COVID-19 and raising the overall productivity level of the economy.

In a nutshell, Latvia's further growth and prosperity depends on the introduction of the latest technologies, the development of new products, and services, as well as the wider use of digital solutions and improved process efficiency. However, the solutions of the problem of labor supply are also important for ensuring faster growth. Investment in human capital is very important. Providing a growing and productive sector with a workforce is critical, which means reviewing existing adult education programs and encouraging a shift of labor from less productive to productive sectors.

## New Impetus for Healthcare Development

In this section, the authors analyse the Latvian healthcare system, which is in the front line in fighting the pandemic. The authors briefly describe the EU response to the pandemic crisis. However, as health policy is a national competence, the analysis focused on the “diagnosis” of Latvia's healthcare sector before and during the COVID-19 crisis. The assessment of the public support of healthcare in 2020–2021 help formulate future challenges and possible scenarios of the healthcare development.

### The EU Response to COVID-19 Pandemic

Like the entire world, the EU was not ready for the pandemic, and the initial response to the crisis looked *ad hoc*. Furthermore, coordination and cooperation between the Member States was initially difficult. When it came to working together, for instance to procure medical supplies, during the early days of the COVID-19 crisis, the EU countries had unilaterally closed their borders and accused each other of hoarding personal protective equipment. The lack of solidarity vis-à-vis Italy in terms of emergency assistance was a culmination of this early trend. The reintroduction of the internal border controls has been uncoordinated at the EU level and justified only by a national security-health policy frame. Another example of pure coordination is the disjointed adoption of lockdowns in the Member States.

In spite of the initial stage of observation, astonishment and uncoordinated or mixed response, the EU managed rather quickly to demonstrate a high degree of adaptability. Without detailed description of the EU response measures (29), even brief summary of actions in the domains of *Vaccination strategy* and *European Health Union* provide a convincing picture of a wide range of unprecedented initiatives that were designed and delivered in a record time.

The Commission has built and implemented a *Vaccination strategy* to provide diversified portfolio of vaccines for EU citizens at fair prices. This strategy, however, came under fire just as it was beginning to deliver (30). Being positioned as a flagship of the European solidarity, the Commission's joint vaccine procurement is being accused by the national authorities of being too bureaucratic and too slow. Deutsch and Wheaton (30) argue that dozens of interviews with diplomats, Commission officials, pharma industry representatives, and national government aides clearly show “how a vaccine strategy that was supposed to be a forceful show of European solidarity, an assertion of the single market's buying power and a moral stand against Trumpian “vaccine nationalism” resulted in a rollout that has left the EU lagging behind the United Kingdom and the United States”. Despite the criticism, deliveries of vaccine doses to Member States have increased steadily since December 2020, and according to the EC information already in August 2021, 70% of adult EU population have been fully vaccinated.

Until the pandemic crisis, health was off the radar in the priorities of the European policy. Health policy was considered a national competence and health issues was considered almost exclusively as the business of the Member States. The European Union's lack of competence in the field of public health already in the first months of crisis created problems, and the COVID-19 pandemic became a catalyst in the acceptance of the leading role of EU in building a health policy. Since the early spring in 2020, health has dominated the media coverage and national and international debates. In November 2020, the Commission has taken the first steps toward building the *European Health Union* by issuing a set of proposals to strengthen the EU's health security framework and reinforce the crisis preparedness. Against this background, in September 2021, the Commission has launched the European Health Emergency preparedness and Response Authority (HERA), a shared resource and mission control center for MS and EU institutions to better prepare for cross-border health emergency threats. Another important initiative is the Pharmaceutical Strategy for Europe, adopted in November 2020. This strategy is aimed at ensuring access to affordable medicines for patients, supporting competitiveness, innovation, and sustainability of the EU's pharmaceutical industry, enhancing crisis preparedness, and diversifying secure supply chains to address shortages in medicines.

The Commission also managed to restore mobility and ensure that the Member States act *in a coordinated way* to both *contain the spread of the virus* and *keep the safe free movement* of people and goods within the EU. It was difficult to imagine that the Commission, a year and a half after the start of the pandemic, would introduce a “European Covid certificate”, thus allowing people to travel without difficulty within the European Union (31). The EU Digital COVID-19 Certificate Regulation came into practice on July 1, 2021.

### The Latvian Healthcare System Before the COVID-19 Crisis

**Figure 10:**
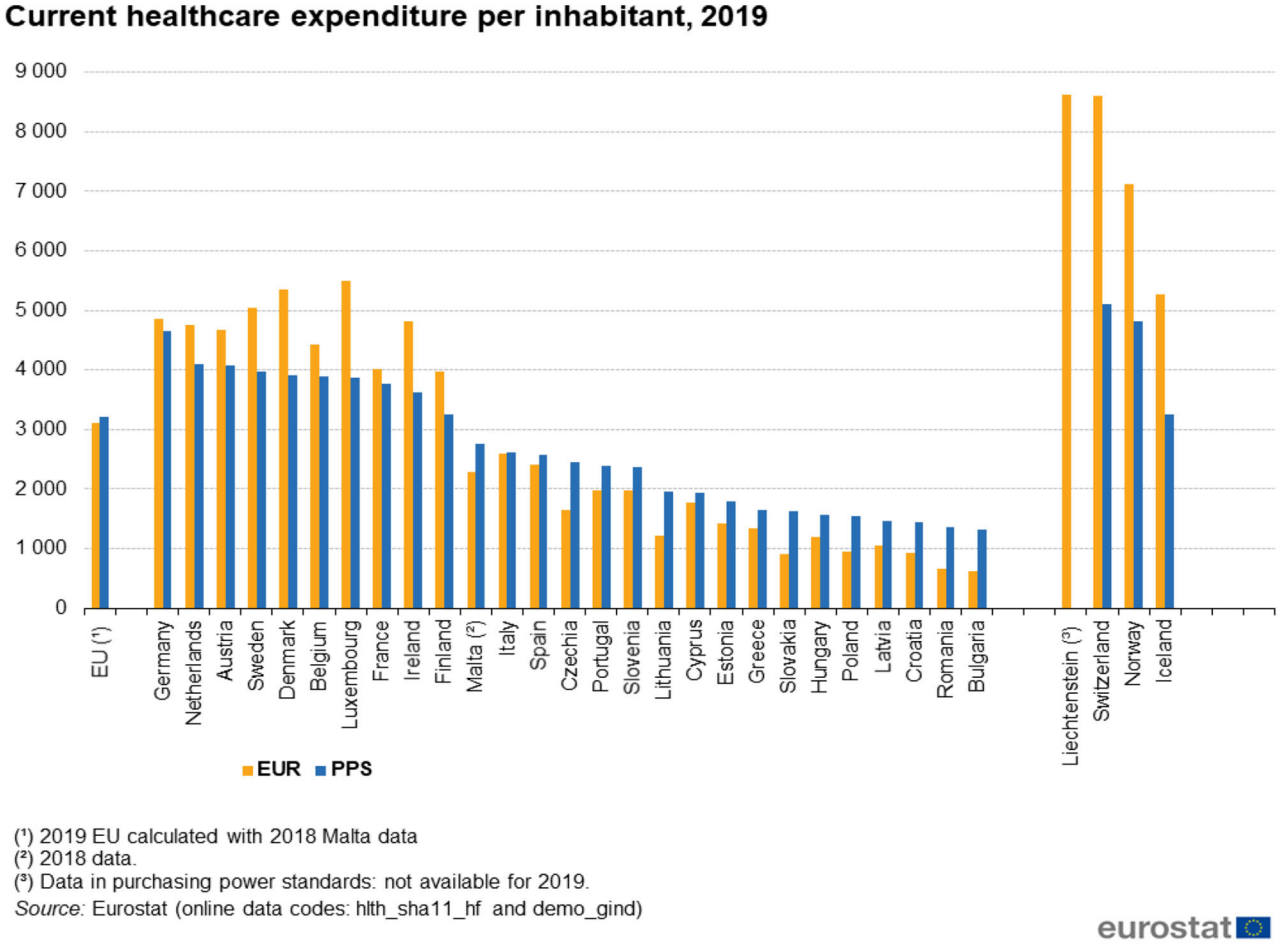
EU current health expenditure per inhabitant, 2019 (in euros) (32). Source: https://ec.europa.eu/eurostat/statistics-explained/index.php?title=Healthcare_expenditure_statistics, accessed: 27/12/2021.

Historically, the Latvian healthcare system has been underfinanced when compared to other EU countries. In 2019, Latvia's current healthcare expenditure per inhabitant was one of the lowest in the EU, only €1,046, while the average level in the EU countries was almost three times higher and reached €3,102 (Figure 10) (32). The comparison is not much better if the healthcare expenditure is adjusted to purchasing the power terms (32).

Furthermore, the picture does not become better if Latvia's current healthcare expenditure is measured in terms of the share of GDP: it accounted for mere 6.6% of GDP in 2019. Only Romania, (5.7%) Hungary (6.4%), and Poland (6.5%) had allocated a smaller share of GDP to the sector (also Luxembourg because of cross-border workers), while the average share in the EU was 9.9% (32).

Since 2014, Latvia, like the two other Baltic States, has steadily increased its current health expenditure at a faster rate that resulted in the cumulative increase of the expenditure of 56% by year 2019. This increase was significantly higher than in the EU (18%). Nevertheless, considering the expenditure level per inhabitant, Latvia has much catching to do, even when compared to its Baltic neighbors (33).

In accordance with the EU classification, there are three main sources of health financing: (i) government schemes, (ii) compulsory contributory schemes and saving accounts, and (iii) other financing agents. Latvia is one of the only four EU countries where compulsory schemes do not exist (32). All the financing has to come either from the state budget or from other (mostly private) sources. That is apparently one of the reasons why the health financing in Latvia is rather low.

**Figure 11:**
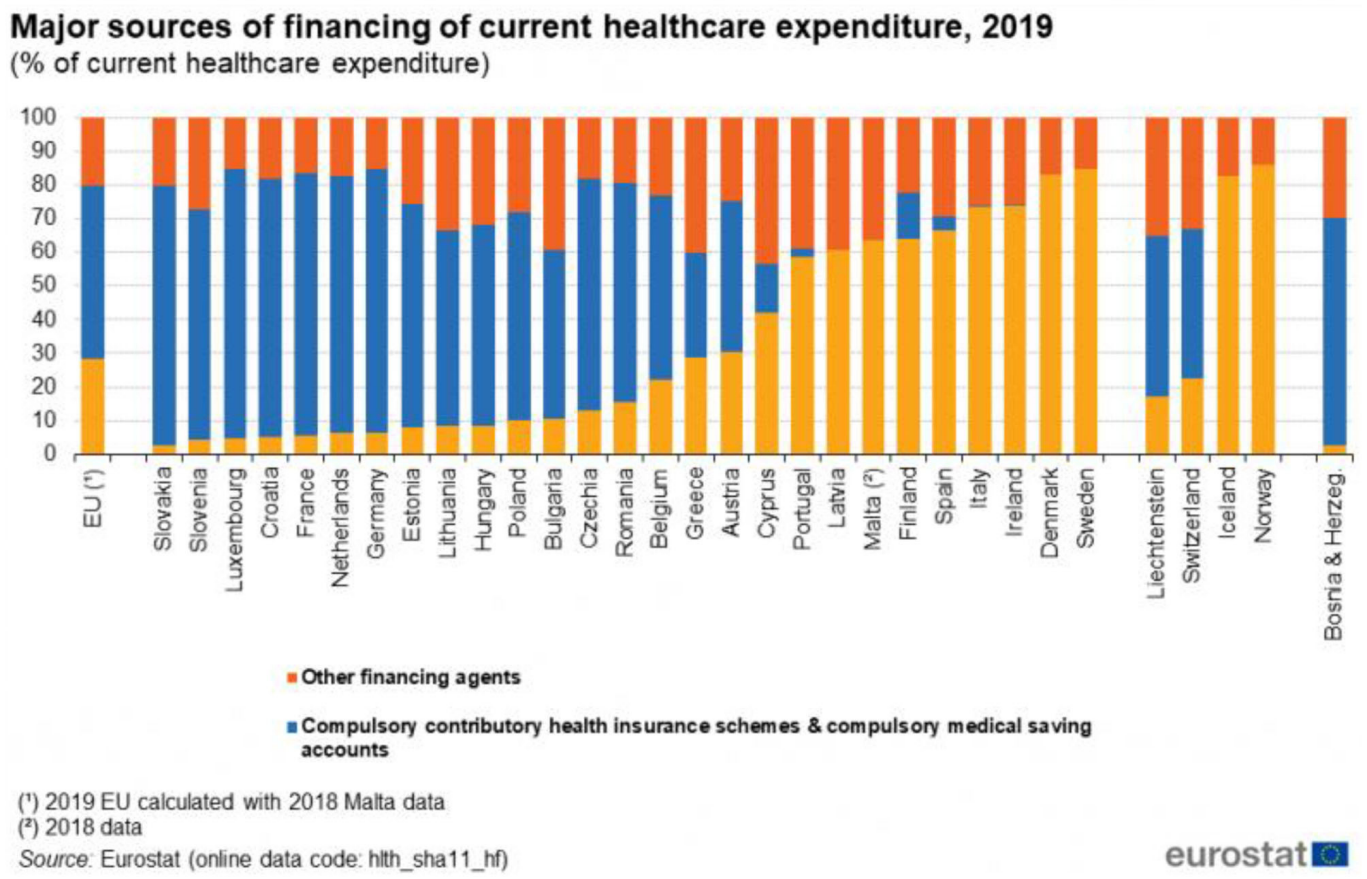
Major sources of financing of current healthcare expenditure in EU countries, 2019 (%) (32). Source: Eurostat https://ec.europa.eu/eurostat/statistics-explained/index.php?title=Healthcare_expenditure_statistics, accessed 27/12/202.

In most of the EU Member States, either government schemes or compulsory schemes/accounts are by far the most important source of healthcare financing. However, since the latter does not exist in Latvia, the two other sources contribute relatively a modest share (60.8%) among the total current healthcare financing. Thus, household out-of-pocket payments, whose share averaged 15.4 % in the EU in 2019, played an important role in case of Latvia, 35.6%, which together with Bulgaria (37.8%), Greece (35.2%), and Malta (34.3%) were the only countries in the EU where out-of-pocket payments accounted for above one third of the total healthcare expenditure. For example, in much wealthier countries like France and Luxembourg, the household out-of-pocket payments accounted for less than one tenth (9.3 and 9.6%, respectively) of healthcare expenditure (Figure 11) (32).

Eurostat data shows that Latvia has one of the highest Gini coefficients in the EU, 35.2 (average in the EU 30.7) in 2019 (34). Only Bulgaria (40.8) and Lithuania (35.4) have a worst ratio. Thus, the excessive reliance on private financing to provide healthcare seems to show the country's ill designed health policy for years that ignores income inequality in the country. This seems at odds with the common accepted EU values and principles of health systems, that universal access to quality healthcare, at an affordable cost to both individuals and society at large, is widely regarded as a basic need.

Data collected in accordance with the COFOG classification (35) prove that despite the lack of compulsory contributory health insurance schemas, the health sector in Latvia received one of the lowest shares of the general government expenditure, 4.2% in 2019, while the average share in the EU were 7.1%.

**Figure 12:**
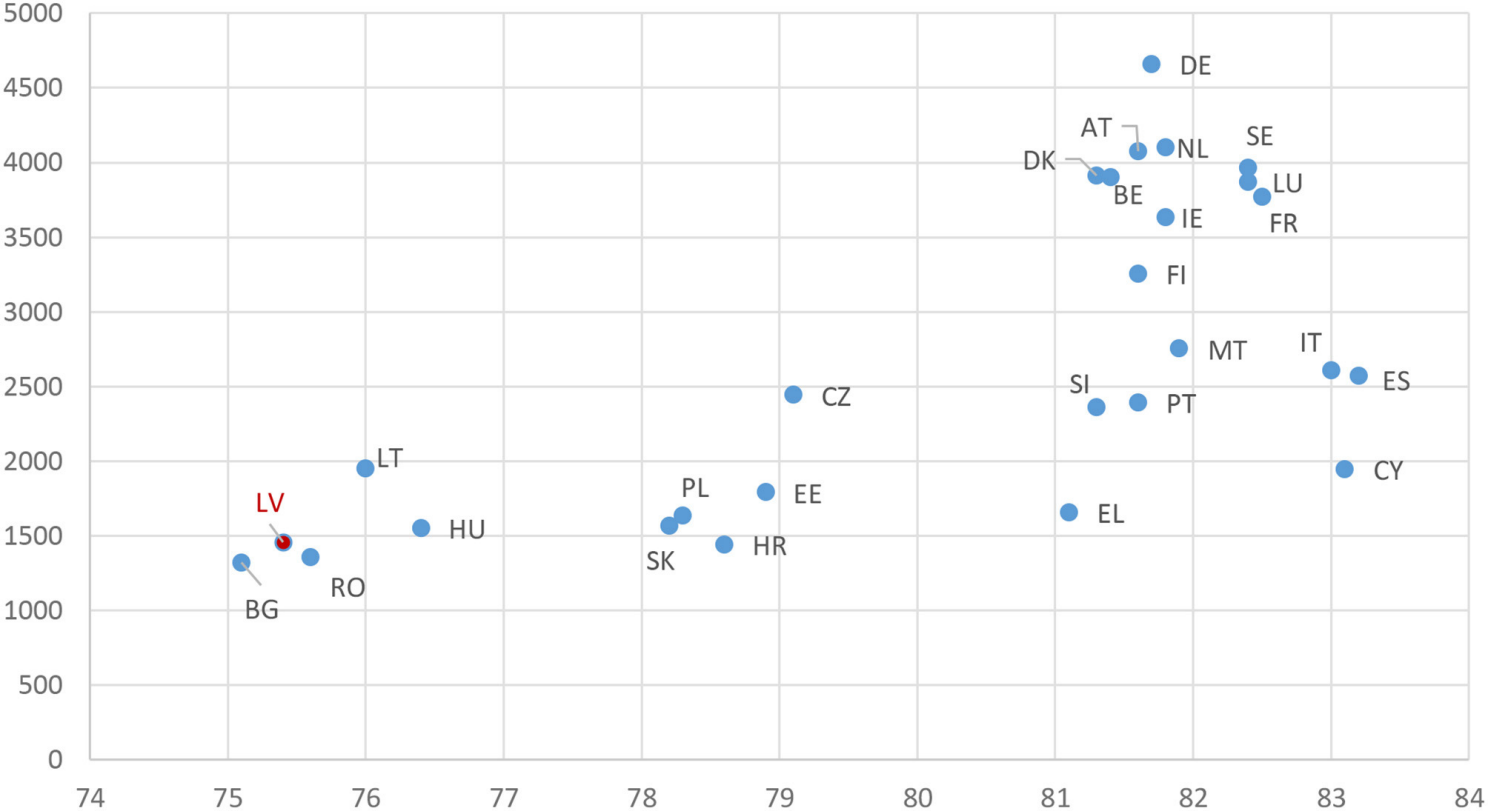
Life expectancy at birth (years) and healthcare expense per inhabitant adjusted with purchasing power (euros) in EU countries in 2019 (33, 36, 37). Source: Eurostat (online data codes hlth_sha11_hf and demo_gind) and WHO statistics.

Regardless of the angle of the analysis, it is clear that the health sector in Latvia had received a relatively modest financing. The apparent negligence of the sector is even more surprising considering the health status of the nation. Life expectancy is considered one of the main health indicators of the nations' health. Figure 12 (33, 36, 37) shows that Latvia has one of the lowest life expectancies in the EU (37) (2019 data) and at the same time has one of the lowest current healthcare expenditures per inhabitant, adjusted for purchasing power standards, despite recent increase in expenditure in comparison to the other EU countries. The correlation between the life expectancy and expenditure is clearly observable, and Latvia's population health status and expenditure levels are lagging compared to the other Eastern Europe countries.

The hypothesis of poor state of health of population in Latvia is supported by other WHO statistics (37): Latvia has the highest maternal mortality ratio in EU, 19 per 100,000 births (data 2017), the second highest tuberculosis incidence of 26 per 100,000, and the lowest healthy life expectancy at birth, of 66.2 years.

To sum up, even though Latvia has a relatively poor status of population health and despite an increase in the healthcare budgetary spending in the recent years, health expenditure level per capita in Latvia remains low in comparison to the other EU countries. Absence of compulsory contributory schemes and relatively small share of government expenditure allocated to healthcare stand out as two major reasons. This conclusion is at odds to the fact that Latvia has one of the highest proportions of the population receiving a pension, over 30% (38) i.e., the large number of pensioners who are the main beneficiary group of effective healthcare did not result in social policies with adequate healthcare financing. The recent EU “State of Health in the EU, Latvia” report (39) echoes our conclusion: “life expectancy in Latvia remains low compared to other EU countries due to the relatively high prevalence of behavioral risk factors, as well as low public spending on health and care accessibility issues”.

The EU Council in its yearly country specific recommendations has persistently advised Latvian governments to pay more attention to its healthcare system (40). In 2014, the Council recommended to “improve the cost-effectiveness, quality and accessibility of the healthcare system”. Later, the EU Council recommendations basically conveyed similar messages: “take action to improve the accessibility, cost-effectiveness and quality of the healthcare system and link hospital financing to performance mechanisms,” (2015) “increase the cost-effectiveness of and access to healthcare, including by reducing out-of-pocket payments and long waiting times,” (2017) “increase the accessibility, quality and cost-effectiveness of the healthcare system,” (2018) and “increase the accessibility, quality and cost-effectiveness of the healthcare system” (2019). Latvia has responded by increasing the healthcare expenditure in the recent years, but the level of adequate expenditure has not been reached (33).

Health workforce shortage is one of the main problems in the Latvian healthcare system. In 2019, the number of practicing doctors in Latvia were 3.3 per 1,000 inhabitants (3.9 on an average in EU). The situation with nurses is even more dramatic, as the number of nurses is about half the EU average (8.4 per 1,000 inhabitants) (39). Another problem is that healthcare workers in Latvia are concentrated in cities, thus limiting the healthcare access to less affluent population in the rural areas. The Ministry of Health of Latvia estimated that at the end of 2019, there was a shortage of 1,500 nurses in Latvia, while to reach the optimal number of nurses, 3,050 more of them are needed (41). The report (41) highlights that the number of working nurses has decreased by 21% over the last ten years and around 40% of nurses are in retirement or close to the retirement age. The Latvian government did respond to the shortage of health workforce shortage: it approved a Conception on “Healthcare system reform” (42) in 2017 that among other things envisioned to increase the average wages for doctors from €1,270 s in 2018 to €2,455 in 2023; and for nurses, from €762 in 2018 to €1,473 in 2023, almost a double increase in 5 years. Despite the initial delay in implementing the healthcare pay reform, the COVID-19 crisis speeded up the pay increase: the 2022 budget provides that the minimal wage of a doctor will be €1,555, and of a nurse is €1,032 (43). There seems to be a high degree of fragmentation and lack of information availability on the pay for health workforce which impedes a full analysis of the pay situation in the Latvian health sector.

### The Impact of COVID-19 Crisis on the Latvian Healthcare System

Like many countries, COVID-19 struck the Latvian healthcare system hard. Even though considering the lack of adequate funding and the initially very slow vaccination speed in Latvia, the cumulative confirmed COVID-19 death rate per million people was not considerably higher in Latvia (2,477) than the average in the EU, which was 2,044 as of January 6, 2022 (44). For example, in the neighboring Lithuania, with the substantially higher health care system financing the number was 2,786. It should be kept in mind that interpretation of these data should be done carefully, as COVID-19, in many instances is not *the* cause of death, but only one of the factors. In addition, there might be different data collection and testing methodologies. Nevertheless, the Latvian healthcare system seems to have responded to COVID-19 crisis adequately, if success is measured by the number of deaths per capita, especially considering the limited allocation of resources and the described problems in the Latvian healthcare system. Achieving the relatively adequate level of death rate is remarkable since Latvia, for a rather long time, lagged in the vaccination rates, in comparison to other EU countries.

As the analysis presented in the first section (Figure 6) (13, 14) shows, compared to many other EU countries, on average, the Latvian people share rather an individualistic value system. This cultural trait partly explains why the vaccination was so slow, especially at the beginning of the vaccination campaign. It follows that the government probably should have used mandatory vaccination at an early stage; however, in this case, it is most likely that the trust in the government would have been significantly eroded.

Like other counties, Latvia responded to COVID-19 crisis with fiscal stimulus and socially protecting measures. Naturally, due to the medical cause of the crisis, the health sector was one of the principal recipients of the additional budgetary funds. In 2020, when the COVID-19 infection rate in Latvia was relatively low, the healthcare system received 12.7% of the total state COVID-19 support. As stated in the Latvia's Productivity Report 2020, “although, overall, Latvia's support program appears modest compared with that of other countries, the study concludes that if we plot State aid intensity against the spread of COVID-19 in the country, the Latvian support program appears to be very proportionate to the spread of the pandemic” (45). In 2021, when the infection rate has worsened significantly, the state support increased almost twice and reached 21.3%. As the share of GDP, government COVID-19 support measures of healthcare system reached 3.3% in 2020 and could reach 8.9% in 2021 (46).

There is no accurate estimate in the planned size of additional financing in 2022 yet, even though substantial investments of about €400 millions in the infrastructure are planned and additional current healthcare expenditure is likely to be allocated due to the rapid spreading of Omicron, it is likely that state support might be substantial. The EU General escape clause, which remains in force in 2022, allows the governments to run budget deficit without mandatory restrictions. The scheduled national elections in Latvia in the autumn of 2022 might also motivate the politicians to allocate an increasing amount of public funds for various causes, including healthcare.

COVID-19 support to health sector has substantially increased its budget allocation share (47). However, despite some progress, the Latvian budget allocation to the sector falls short on EU levels (35). In addition, it is likely that in response to COVID-19 crisis, other EU Member States have also increased the health financing share, and the gap between Latvia and EU average would not be bridged.

Investment in health care related to the containment of the COVID-19 pandemic has also permanently boosted the sector. In accordance with the Ministry of Health information (48) by September 2021, €123.6 million or around 25% of the total budget funds allocated to prevent and fight COVID-19 infection was used for investments in the facilities and infrastructure or medical equipment. These investments apparently will permanently bolster the capacity of the health sector even after the COVID-19 crisis.

### Future Scenarios in the Development of Healthcare System

COVID-19 crisis has increased the capacity of the Latvian health sector, both in terms of investments in infrastructure and equipment, and increasing the pay to the healthcare staff. But it remains to be seen how lasting will be the effect of an increased capacity and whether it will be sufficient. The World Health Organization has forecasted that COVID-19 pandemic will transform into an endemic state by next winter. The impact of the endemic, with new variants to emerge likely, means that the ongoing pressure on the healthcare system, even amid the costs will be less severe.

The budget law 2022 (49) provides a 7.1% increase in expenditures for the Ministry of Health in comparison to the budget law of 2021. Considering the latest inflation forecast of 6.1% by the Bank of Latvia (50), it is a very modest increase. For instance, the increase of remuneration is only 4.2% i.e., below the expected inflation rate. On the contrary, capital expenditure in the health sector in 2022 will increase significantly and reach €21.2 million, three times of 2021 level. Of course, any further COVID-19 wave will require extra spending for health sector, including the remuneration for the sector staff. However, currently investments into the infrastructure seem to be the higher priority of the government as compared with investments in the human resources.

It is difficult to forecast the health expenditure beyond 2022. It should be noted that it is expected that some kind of fiscal restrictions will be in force starting by 2023, which should restrict the government expenditure. Therefore, the risk to return to the precrisis underfinancing of health care is rather high.

### Lessons Learned

The shape and the functioning of national economies has changed disruptively as a consequence of the COVID-19 shock. Government's institutions need to respond efficiently to facilitate economic growth and reduce the negative effects of structural changes in labor markets across sectors and regions. A fiscal policy regime change is underway, which will reduce the scope of budget spending.

Small economies often recover quickly from economic shocks. As far as Latvia is concerned, many causes of the governance problems, a weak coordination of activities between the public institutions and economic sectors, lack of regulatory effectiveness assessment, and insufficient learning from identified policy shortcomings, were inherited from the prepandemic period. While the pandemic spectacularly exposed the problems in the public governance, it also presented an opportunity to break from the bad institutional inertia and to close the existing deficiencies.

A public opinions poll carried out in the framework of this study showed that the Latvian people have been pretty accepting the work of the public authorities during the pandemic. This acceptance declined during the later stages of the pandemic; nevertheless, it stayed relatively high considering the lack of foresight, and the decisive and consequent actions from the government.

The pandemic has unlocked a significant activity from the Latvian government; however, there is a good ground to share the concern about the sustainability of this activity in the postpandemic period. Without a more dynamic and efficiency-oriented institutional environment in the public sector, a sustainable postpandemic recovery will not be possible. More thorough attention to learning from own policy mistakes and improved inter-institutional coordination are warranted. These would embody the first essential steps in the case of Latvia, as put by OECD, “to build a deeper and more entrenched capability to allow for transformation not only in response to a crisis, but a transformation that meets longer-term needs and that fits with the values that the government aspires to (51)”.

One of the reasons for Latvia's relatively slow convergence of GDP per capita to the level of highly developed EU countries is its low productivity. Proproductivity policies can be a particularly challenging task. There is neither a silver-bullet solution, nor a standard set of reforms that can be implemented in the same way in every EU country.

The competitive advantages of the Latvian economy are mainly based on technological factors, improvement of production efficiency, innovation, and digitalisation. The crisis caused by the COVID-19 pandemic is a catalyst for more rapid change (digitalisation, teleworking, etc.). However, the fundamentals of productivity remain unchanged and relate to investment in human capital, investment in new technologies and capital intensity, the ability to integrate into global value chains and increase export potential, innovation, the development of new products, services, and methods.

The pandemic crisis highlighted EU problems in coordinating the healthcare policies in the Member States and caused an adequate EU response. However, in general, health policy is a national competence and health issues, almost exclusively, are the business of the Member States. In Latvia, before the crisis, the healthcare sector suffered from underfinancing and lack of reforms. During crisis, despite enormous pressure on the healthcare system, there have been also positive implications for the sector, which for decades was underfinanced. The sector finally got the attention of the political leaders and received significant state support, which allowed to raise the wages of the medical staff and to invest in medical equipment. These measures helped maintain healthcare services at a reasonable level during the COVID-19 crisis.

However, future scenarios of the healthcare development are unclear. Returning fiscal constraints, which currently are put on hold, will affect the budgetary spending. Assuming that in the years to come, various needs have to be accommodated, the healthcare, which since 2020 experienced additional budget support, can be considered as a lower-priority sector compared to other urgent needs. Without the substantial increase of productivity and acceleration of growth, it would be difficult to rely on sufficient health financing from the state budget. Further increase in the capacity of the healthcare system should be among the priorities of the government as a substantial part of the social policy. The Latvian society is aging and the healthcare system needs a comprehensive reform rather than the *ad hoc* “cosmetic” improvement.

To finish on a positive note, the COVID-19 has strengthened the incentives around business investment in technologies and new business models (automation, digital, etc.). To the extent that accelerated investments from private and public sources including the EU funds will be effective (which we think is plausible), it is possible that the increased productivity in 2022 and beyond will foster the economic growth, and in turn, strong growth will help increasing the budgetary spending in the priority sectors.

## Data Availability Statement

The raw data supporting the conclusions of this article will be made available by the authors, without undue reservation.

## Author Contributions

IŠ contributed to conception, design of the study, wrote abstract, introduction, introductions to the chapters, and conclusions. AA applied a special theoretical framework for assessment of effectiveness of Latvian institutions, designed a special survey for assessment of public perceptions, applied mathematical analysis to collected data, and wrote the first chapter of the manuscript. OB performed statistical and shift share analysis for evaluation of macroeconomic trends and future scenarios, and wrote the second chapter of the manuscript. NM applied statistical analysis and analysed policy documents related to public spending on healthcare, and wrote the third draft of the manuscript. All authors contributed to manuscript revision, read, and approved the submitted version.

## Funding

The research was supported by the National Research Programme Latvian Heritageand Future Challenges for the Sustainability of the State Project Challenges for the Latvian State and Society and the Solutions In International Context (Interframe-LV, Project No.VPP-IZM-2018/1-0005).

## Conflict of Interest

The authors declare that the research was conducted in the absence of any commercial or financial relationships that could be construed as a potential conflict of interest.

## Publisher's Note

All claims expressed in this article are solely those of the authors and do not necessarily represent those of their affiliated organizations, or those of the publisher, the editors and the reviewers. Any product that may be evaluated in this article, or claim that may be made by its manufacturer, is not guaranteed or endorsed by the publisher.
